# Efficacy, Safety and Outcomes of the Laparoscopic Management of Cesarean Scar Ectopic Pregnancy as a Single Therapeutic Approach: A Case Series

**DOI:** 10.3390/jcm12247673

**Published:** 2023-12-14

**Authors:** Georges Salem Wehbe, Inesse Ait Amara, Michelle Nisolle, Dominique A. Badr, Marie Timmermans, Stavros Karampelas

**Affiliations:** 1 Department of Obstetrics and Gynecology, University Hospital Brugmann, Free University of Brussels, 1020 Brussels, Belgium; georges.salemwehbe@chu-brugmann.be (G.S.W.); inesse.aitamara@chu-brugmann.be (I.A.A.); dominiquebader@hotmail.com (D.A.B.); 2 Department of Obstetrics and Gynecology, Hospital the Citadelle, University of Liege, 4000 Liege, Belgium; michelle.nisolle@chuliege.be (M.N.); marie.timmermans@chuliege.be (M.T.)

**Keywords:** cesarean scar pregnancy, laparoscopic cesarean scar pregnancy excision, isthmocele, residual myometrium thickness

## Abstract

A standardized consensus for the management of cesarean scar pregnancy (CSP) is lacking. The study objective is to evaluate the efficacy, safety and outcomes of the laparoscopic management of CSP as a single therapeutic surgical approach without being preceded by vascular pretreatment or vasoconstrictors injection. This is a retrospective bi-centric study, a case series. Eight patients with a future desire to conceive underwent the laparoscopic treatment of unruptured CSPs. Surgery consisted of “en bloc” excision of the deficient uterine scar with the adherent tissue of conception, followed by immediate uterine repair. The data collected for each patient was age, gestity, parity, number of previous c-sections, pre-pregnancy isthmocele-related symptoms, gestational age, fetal cardiac activity, initial β-human chorionic gonadotropin levels, intra-operative blood loss, blood transfusion, operative time and the postoperative complications, evaluated according to Clavien–Dindo classification. The CSP was successfully removed in all patients by laparoscopy. The surgical outcomes were favorable. All patients with histories of isthmocele-related symptoms reported postoperative resolution of symptoms. The median residual myometrium thickness increased significantly from 1.2 mm pre-operatively to 8 mm 3 to 6 months after surgery. The laparoscopic management seems to be an appropriate treatment of CSP when performed by skilled laparoscopic surgeons. It can be safely proposed as a single surgical therapeutic approach. Larger series and further prospective studies are needed to confirm this observation and to affirm the long-term gynecological and obstetrical outcomes of this management.

## 1. Introduction

Ectopic pregnancy (EP) is a serious complication in early pregnancy. Non-tubal ectopic pregnancies, which account for less than 10% of all extra-uterine pregnancies, may implant in the peritoneal or abdominal cavity, the ovary, the cervix or in the scar of a previous cesarean section (CS) [1,2,3,4]. Over recent decades, the rate of delivery by CS increased significantly in both developed and developing countries. Therefore, despite that cesarean scar pregnancy (CSP) is the least frequent type of ectopic pregnancy, its incidence is rapidly increasing [5,6,7]. A standardized consensus for the management of CSP is lacking. The laparoscopic management of CSP has been recently gaining interest as a fertility-preserving surgery [8,9,10]. Fu et al. found laparoscopy superior to laparotomy for treating CSP. However, in this series, different methods of vascular pretreatment, such as uterine artery embolization (UAE) or temporary/permanent arterial occlusion, have been applied. Moreover, dilatation and curettage (D&C) of the uterus was performed before the lower uterine scar was cut [11]. Few other studies were published regarding the use of laparoscopy for the management of CSP [12,13,14]. Wang et al. evaluated the feasibility and efficacy of laparoscopy for patients with ectopic pregnancies in unusual locations. Twenty-two patients with CSP were treated laparoscopically after intramyometrial injection of diluted vasopressin at one or more sites before uterine incision [12]. There are no studies evaluating the feasibility, safety, outcomes and efficacy of the laparoscopic management of CSP as a single therapeutic approach without being combined with any other strategy or vascular pretreatment. In this study, we report our experience of the past 4 years in the treatment of eight patients who underwent laparoscopic resection of CSP solely as a fertility-preserving option.

## 2. Materials and Methods

This is a retrospective bi-centric study (case series) performed between December 2019 and January 2023 in the Departments of Obstetrics and Gynecology of the CHU Brugmann in Brussels and Hôpital de la Citadelle in Liège, Belgium. The study was approved by the Hospitals Ethics Committees under the reference numbers CE2022/276 and B412201836328, respectively. In this series of patients, we included women with a future desire to conceive, who agreed to be treated laparoscopically for a current diagnosis of unruptured cesarean scar ectopic pregnancy.

The inclusion criteria for this study were as follows: (1) Patient between 18- and 45-years-old, (2) a history of at least one lower uterine segment cesarean delivery, (3) a positive serum beta-human chorionic gonadotropin (β-hCG), (4) a diagnosis of CSP performed by transvaginal ultrasound.

A diagnosis of CSP was made when the following sonographic criteria were met: (1) uterine vacuity, with a well-visualized endometrium along its entire length, (2) vacuity of the cervical canal, which is not in contact with the gestational sac, (3) visualization of a gestational sac at the lower uterine segment over the previous hysterotomy scar/niche with a yolk sac and/or a fetal pole with or without heartbeat, (4) decreased myometrial thickness or disappearance of the myometrium between the gestational sac and the uterine serosa or the bladder with or without protrusion of the gestational sac through the muscular defect, (5) detection of peri- trophoblastic blood flow using Doppler ultrasound, (6) absence of a “sliding sign” when pressure is applied to the lower uterine segment using the transvaginal ultrasound probe. Indeed, in a miscarriage, the gestational sac slides against the endocervical canal, but does not do so in CSP [15,16].

Only patients with type II CSP, defined as a gestational sac implanted on the cesarean scar with protrusion towards the myometrium, were treated laparoscopically [15].

Since significant association between cesarean scar ectopic pregnancy and uterine scar defect has been reported, screening for isthmocele-related symptoms occurring before pregnancy was performed. Patients were considered symptomatic when they had one or more of the following complaints: (1) abnormal uterine bleeding (AUB) reported as ≥ 2 days of intermenstrual bleeding or spotting, (2) chronic pelvic pain (CPP), described as continuous or intermittent pain in the anatomical area below the umbilicus and between the hips, associated or not with dyspareunia for at least 6 months and not otherwise explained, (3) secondary infertility not otherwise specified [10,17,18].

The treatment proposed was the “en bloc” laparoscopic excision of the deficient uterine scar with the adherent gestational sac or trophoblastic tissue, followed by an immediate uterine reconstruction [10]. Prior to surgery, a complete medical history and clinical examination were performed for every patient included in the study. Serum β-hCG determination was obtained, as well as a transvaginal ultrasonography to confirm the diagnosis. All patients were informed about the risks and benefits of the proposed treatment, the potential need to convert to laparotomy, the risk of intra-operative urinary tract injury, the probability of massive bleeding and blood transfusion and the possibility of postoperative infection and intra-abdominal/pelvic adhesion formation. The patients were systematically asked to use adequate contraceptive methods postoperatively for at least a period of 6 months to prevent pregnancy before a proper healing of the uterine scar.

There is lack of strong evidence in the literature regarding the safety or the favorable maternal and neonatal outcomes of the trial of labor or vaginal delivery after CSP and uterine scar defect excision and repair. Therefore, patients were counselled to deliver by CS at 39 weeks during subsequent pregnancies. A proper informed consent was signed prior to surgery by all women who accepted the proposed management. All the procedures were performed by one gynecologic surgeon (SK) with more than five years of surgical experience in the field of minimally invasive gynecological surgery and particularly in uterine niche excision and repair performed. All the surgical interventions were performed using the same surgical technique described in detail, as follows:

− After CO_2_ pneumoperitoneum using conventional techniques, a laparoscope was inserted to explore the anterior wall of the uterine isthmus to determine the CSP volume, the relationship between the uterine scar and the bladder and the presence of adhesions.
− In case of adhesions of the uterine scar with the anterior abdominal wall, adhesiolysis was performed.
− The vesico-uterine fold was dissected, and the bladder was detached from the CSP and pushed caudally in order to completely expose the ectopic pregnancy.
− Using monopolar energy or cold scissors, the deficient uterine scar was excised “en bloc” along with the adherent trophoblastic tissue or gestational sac. The myometrial incision was systematically performed at 3–4 mm from the distal and proximal parts of the gestational sac. This way, the perforation and the entrance into the tissue of conception zone and the resulting heavy bleeding were avoided.
− The cesarean scar was completely opened from the right towards the left angle of the defected scar. Excision of the fibrotic tissue from its edges was performed, reaching healthy myometrium.
− A Hegar probe was inserted into the cervix before the closure of the uterine defect, so that the continuity of the cervical canal with the uterine cavity was preserved.
− Three separate figure-of-eight sutures using monofilament absorbable thread composed of poliglecaprone 25 (ETHICON 0-Monocryl) were placed in order to close the deepest layer of the scar, along with the endometrium.
− A double-layer scar closure was performed by applying a second superficial layer of running suture using a monofilament thread (ETHICON 2/0-Monocryl).
− A uterine anterior suspension procedure consisting of a retroperitoneal lateral suspension of the round ligament to the external oblique abdominal muscle aponeurosis was performed in the presence of a retroverted uterus. This step was performed in order to relieve the tension on the previously applied sutures, since a persistent uterine retroversion may impair wound healing and predispose the patient to the formation or recurrence of scar defects [10,17,18].

**Figure 1:**
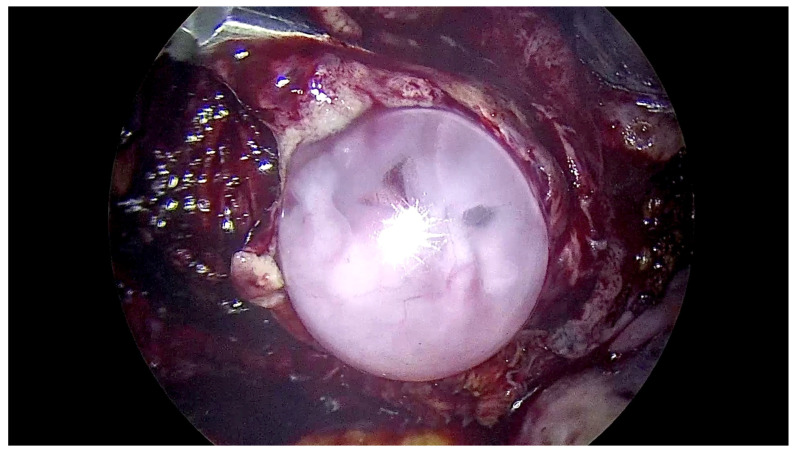
Intraoperative laparoscopic view of an intact gestational sac resected “en bloc” with the defected myometrium.

**Figure 2:**
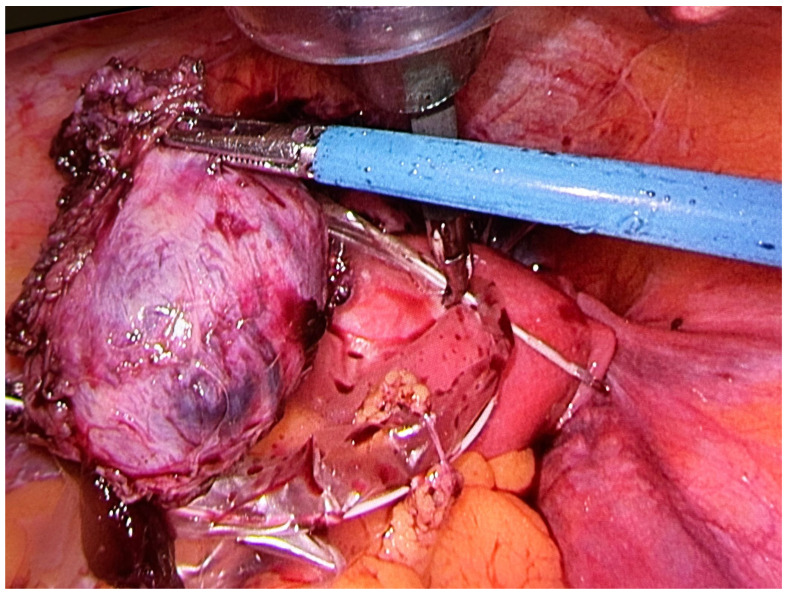
Complete resection of the defected scar, along with the ectopic pregnancy, respecting the principle of 3–4 mm margin of healthy adjacent myometrium to prevent severe intra-operative bleeding.

Figure 1 and Figure 2 correspond to intraoperative laparoscopic views of an intact gestational sac resected “en bloc” with the defected myometrium.

Pre-operatively, the diagnosis of CSP and the assessment of the residual myometrium (RM) were performed by certified sonographers using transvaginal ultrasound (TVUS). The diagnostic criteria described above were systematically applied. The RM measurement was performed on the sagittal plane (thinnest RM). The postoperative uterine assessment was carried out using saline infusion sonohysterography (SIS) 3 to 6 months after surgery by certified sonographers.

For each patient, the following data were collected after a thorough retrospective chart review: age, gestity, parity, number of previous c-sections, pre-pregnancy isthmocele-related symptoms, gestational age at time of diagnosis of CSP, fetal cardiac activity, initial β-hCG (IU/L), presenting pre-operative symptoms, intra-operative blood loss (mL), blood transfusion, operative time (minutes) and postoperative complications, evaluated according to Clavien–Dindo classification [19].

Statistical analysis was performed using R software version 4.2.2 (www.r-project.org). Continuous variables were summarized as median (interquartile range [IQR]), while categorical variables were expressed as numbers (frequency). Wilcoxon matched-pair signed-rank test was used to compare the median values of the paired samples. The following R package was used: “ggpubr”. Statistical significance was set at *p* < 0.05.

## 3. Results

Between December 2019 and January 2023, a total of eight women were eligible and gave informed consent to undergo the proposed laparoscopic management of CSP. The baseline clinical characteristics of the study population are summarized in Table 1.

The diagnoses of the cesarean scar ectopic pregnancies were made between the 5th and 9th weeks of gestation, with a median β-hCG level on admission of 21,619.5 IU/L (IQR: 1941.8–59,356.3). All patients upon admission had vaginal spotting/bleeding associated with mild abdominal discomfort. The median age of the patients was 36 years (IQR: 34.5–39.8). Seven out of eight patients had a history of at least two previous cesarean sections.

The ectopic gestational sac, along with its content, such as tissue of conception and/or embryo, were successfully and completely removed in all patients. None of the patients operated upon had retention of residual trophoblastic tissue on follow-up SIS. In our study population, four patients (50%) reported isthmocele-related symptoms occurring before pregnancy. After removal of the ectopic pregnancy, followed by the immediate repair of the cesarean scar defect, all of them (100%) reported resolution of their isthmocele-related symptoms.

Five patients had desires for future pregnancies, of whom three (60%) had spontaneous pregnancies at least 6 months after surgery after stopping contraception.

The surgical outcomes are summarized in Table 2.

The median operative time was 150 min (IQR: 105.5–163.5). The median hospital stay was 24 h (IQR: 24–33). Regarding blood loss, only one patient out of eight (1/8: 12.5%) had blood loss estimated at 300 mL. The other patients had losses estimated at less than 50 mL. No patient required pre- or postoperative transfusions. There was no conversion to laparotomy, and we did not identify any postoperative complications.

**Figure 3:**
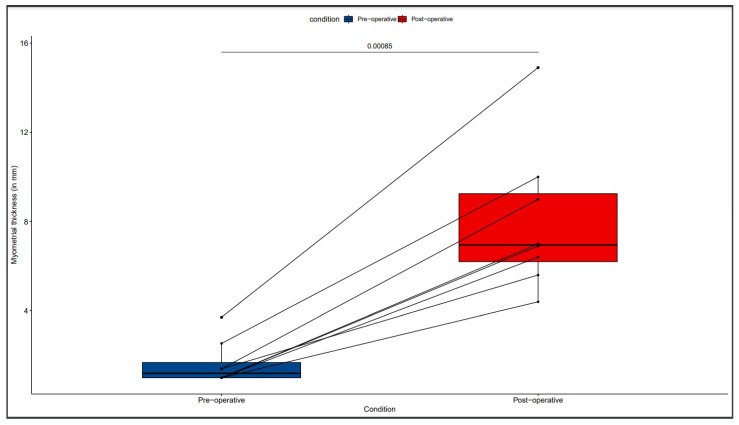
Measurements of the residual myometrial thickness pre-operatively and 3 to 6 months after cesarean scar pregnancy excision and myometrial repair in the study population (N = 8). The residual myometrial thickness increased significantly after surgery (*p*-value: 0.008).

The median RM thickness increased significantly from 1.2 mm pre-operatively (IQR: 1–2.3) to 8 mm (IQR: 5.8–9.8) 3 to 6 months after surgery (*p*-value: 0.008) (Figure 3).

## 4. Discussion

In this manuscript, we describe a successful management of CSP. However, in the literature, there is a lack of clear guidelines or algorithms for the management of CSP. At least 10 different therapeutic strategies are currently described as fertility-preserving options, which makes treatment selection more challenging [9,10]. To our knowledge, we are the first to share our experience regarding the feasibility, safety, outcomes and efficacy of the laparoscopic management of CSP as a single therapeutic approach without being combined with any other strategy or vascular pretreatment.

The laparoscopic management of CSP is gaining more and more interest as a fertility-preserving surgery. This approach seems to be associated with high success rate, low complication rate, shorter hospital stay and faster recovery time [8,9,10,11,20].

The report of Fu et al. revealed that laparoscopic surgery preceded by dilatation and curettage and coupled to UAE or temporary/permanent arterial occlusion is superior to laparotomy for treating CSP. A total of 278 patients were included of whom 121 and 157 were treated with laparoscopy or laparotomy respectively. The intra-operative bleeding, the transfusion rate and the duration of hospital stay were lower when the patient was managed by laparoscopy. Despite the fact that this study included one of the largest populations of patients treated by laparoscopy, conclusions are limited by the retrospective design, the single center experience and the reliance on heterogeneous clinical data not originally collected for research purposes [11].

Kathopoulis et al. found that out of 331 cases treated by laparoscopy, only one case required conversion for severe bleeding. Since the risk of hemorrhage is the most feared complication while operating CSP, several preventive strategies are used such as permanent or temporary ligation of the uterine arteries or local injection of various vasoconstrictor agents such as vasopressin before the myometrial incision [20].

The use of sub-serosal injection of vasoconstrictors such as vasopressin before uterine incision may be associated with severe adverse events such as bronchospasm, bradycardia, pulmonary oedema and even cardiac arrest [21,22,23]. In addition, the time limited effect of vasoconstrictor agents or other temporary vascular strategies might temporarily mask an intra-operative bleeding. Therefore, it might give the surgeon a false reassurance which can lead him to end the surgery before achieving a permanent control of hemostasis.

In order to perform a safe uterine artery clamping, the surgeon must have advanced surgical skills and deep knowledge of the retroperitoneal anatomy. The identification and isolation of the uterine artery from the surrounding structures is often challenging especially in the context of hypervascularised gravid uteri. This surgical step lengthens the operating time and may increase the risk of severe hemorrhage as well as mechanical or thermal ureteral injury. Moreover, there is a significant risk of inadvertent vascular injury while removing the hemoclips at the end of the procedure.

Both medical and surgical strategies of vascular pre-treatment might compromise the vascularisation of the reconstructed uterine myometrium. Although temporary, this iatrogenic ischemia may alter the quality of the uterine wound healing and favor niche formation and/or recurrence of CSP [24].

For all the above listed reasons, we adopted the laparoscopic management of CSP as a simplified therapeutic approach without being combined with any other strategy or vascular pretreatment. In our series of eight patients, the surgical outcomes were favorable. The mean hospital stay was 28.5 h and did not exceed 48 h. This suggests a rapid recovery of the study patients. Conversely, we found a hospital stay ranging from 1 to 9 days in other reports when laparoscopic management was combined with other strategies, such as vascular pretreatment and/or endo-uterine aspiration [20]. This favorable outcome seen in our study population may be the result of our simplified surgical approach.

Ectopic pregnancies account for 1–2% of all pregnancies in the general population. The estimated incidence of cesarean scar ectopic pregnancy, which accounts for only 6% of all ectopic pregnancies, is 1:1800 to 1:2216 pregnancies in women with at least one cesarean delivery [3]. CSP is a relatively rare clinical condition. Moreover, only patients with type II CSP were selected as candidates for the laparoscopic treatment. This fact explains the small number of patients (N = 8) who were eligible for inclusion in the analysis over a period of 4 years of clinical practice. Such a small number does not allow surgeons to achieve the advanced surgical skills needed to safely perform the proposed technique and complete its learning curve. Severe intra-operative bleeding is a common complication faced during the surgical management of CSP. This risk might be a main cause of surgical morbidity and/or mortality, especially when the intervention is performed by unskilled surgeons. As shown in the report of Karampelas et al., published in 2021, the same surgical team who performed all the procedures as a management of CSP previously acquired advanced surgical skills and deep experience in the laparoscopic isthmocele excision and repair as a treatment of symptomatic uterine scar defects. These conditions share common clinical and anatomical characteristics. Therefore, their respective surgical treatments share common steps. We believe that in the CSP surgical management, the key clues to prevent bleeding during surgery are the adequate exposure of the CSP and the “en bloc” resection of the defected scar along with the ectopic tissue of conception. This can be achieved by incising the defected myometrial scar 3–4 mm away from the gestational sac. The anatomical limits of the ectopic pregnancy are easily recognized intraoperatively following dissection of the vesico-uterine fold and the subsequent protrusion of the gestational sac.

A main advantage of our surgical strategy is that it was an immediate and definite treatment of CSP. We did not identify any cases refractory to treatment, and long-term monitoring of β-hCG levels was unnecessary. Conversely, a long follow-up period is mandatory following other medical or combined medical and hysteroscopic approaches [25]. This advantage of the laparoscopic approach optimizes the management of non-compliant patients with CSP.

Significant association between CSP and uterine scar defect has been reported. In their report published in 2017, Pan and Liu found a cesarean scar defect in up to 70% of patients after hysteroscopic removal of the ectopic tissue of conception [26]. This observation suggests that CSP is a strong indicator of a pre-existing symptomatic or asymptomatic isthmocele. Another advantage of the laparoscopic approach is that this technique allows not only the evacuation of the ectopic products of conception but also the beneficial correction of the defect and the myometrial reinforcement. It would be reasonable to think that this might reduce the risk of recurrence of CSP by eliminating a major predisposing risk factor [15,26]. In our series, the mean RM thickness increased significantly 3 to 6 months after surgery. This might theoretically be beneficial for the reduction of the risk of uterine rupture during a future pregnancy. We observed complete resolution of symptoms in 100% of the patients with isthmocele-related complaints before pregnancy. These results are compatible with those found by Karampelas et al., Vervoort et al. and Donnez et al., who used the same surgical procedure as a treatment of symptomatic uterine scar defects in women with RM < 3–5 mm and future desire for pregnancy [10,17,18].

The association between cesarean scar defect and secondary infertility is well known. The accumulation of intrauterine fluid and blood in the isthmocele cavity seems to negatively affect the cervical mucus, sperm quality/transport and embryo implantation [27,28]. The results regarding the effect of laparoscopic resection of the isthmocele are promising, and high rates of fertility restoration following surgery were observed. In their study, Karampelas et al. found that ten out of twelve patients (83.3%) with histories of secondary infertility conceived spontaneously after surgery [10]. Vervoort et al. confirmed the disappearance of the accumulated intrauterine fluid and blood in the majority of women after surgery [18]. Verberkt et al., in a retrospective cohort study including 60 patients, found the following pregnancy rates after initial CSP treatment: 80% after expectant management, 62.5% in patients treated with methotrexate, 86.7% in patients who underwent endo-uterine aspiration and 88.9% in patients managed laparoscopically with resection of the scarring uterine defect [29].

As we mentioned previously, different therapeutic strategies have been described as fertility-preserving options. In our patient population, the median RM thickness was 1.2 mm pre-operatively, which is largely inferior to the 2.05 and 3 mm cutoffs published by Zhang et al. and Donnez et al. This demonstrates the benefits of a laparoscopic approach to avoid the risk of uterine perforation and bladder injury [17,30].

The main limitation of our study was the small number of patients who were eligible for inclusion in the analysis (N = 8). Nevertheless, we were able to identify a trend in the outcomes that seems to correlate with data from the literature. Another drawback was that the time of follow-up in this study was limited. It would be interesting to gain more long-term data, mainly regarding the obstetrical outcomes of patients undergoing this form of treatment.

## 5. Conclusions

Our study findings suggest that laparoscopic management seems to be an appropriate fertility-preserving approach for the treatment of CSP when performed by skilled laparoscopic surgeons. It can be safely proposed as a single and effective therapeutic approach without being combined with any other strategy or vascular pretreatment. Larger series and further prospective studies are required to confirm this observation and to affirm the long-term gynecological and obstetrical outcomes of this management.

## Figures and Tables

**Table 1:** Baseline clinical characteristics of the study population.

Patient	Age (years)	Gestity	Parity	Previous CS	Pre-Pregnancy Symptoms	Gestation Age Upon Admission (weeks)	Serum β-hCG Level Upon Admission (IU/L)	Pre-op RM (mm)	Post-op RM at 1 month (mm)	Post-op RM at 3–6 month (mm)	Spontaneous Pregnancy after Surgery
1	34	3	3	3	Asymptomatic	9	81,528	1	NA	7	+
2	42	3	3	3	CPP	5	1469	1	7.7	6.4	−
3	40	2	2	2	Asymptomatic	5	1576	1.4	NA	5.6	−
4	36	2	2	2	Asymptomatic	7	63,447	1	8.2	4.4	+
5	36	4	3	1	CPP	6	47,084	1.4	10	9	+
6	33	6	3	3	CPP	7	3431	1	NA	6.9	−
7	36	6	3	3	CPP	6	39,808	3.7	14.6	14.9	−
8	39	4	2	2	Asymptomatic	5	3039	2.5	NA	10	−

Note: β-hCG = beta-human chorionic gonadotropin; CPP = chronic pelvic pain; CS = cesarean section; IU = international unit; L = Liter; RM = residual myometrium; NA = not available; mm = millimeter; (+) = YES; (−) = NO.

**Table 2:** The surgical outcomes in terms of operative time, hospital stay, intra-operative blood loss, need for blood transfusion and postoperative complications, according to Clavien–Dindo classification.

Variable	Study Population (N = 8)
Operative time (min)	150 (105.5–163.5)
Hospital stay (hours)	24 (24–33)
Intra-operative Blood loss (<50 mL)	7/8 (78.5%)
Transfusion	0 (0%)
Postoperative complications (Clavien–Dindo classification)	0 (0%)

Note: Continuous variables are represented as median (interquartile range IQR), whereas categorical variables are represented as number (frequency).

## Data Availability

The data presented in this study are available on request from the corresponding author.

## References

[1] Lurie S. (1992). The history of the diagnosis and treatment of ectopic pregnancy: A medical adventure. Eur. J. Obstet. Gynecol. Reprod. Biol..

[2] Ramadan M.K., Kharroubi M., Bou-Ghanem R., Badr D.A. (2020). Bilateral Tubal Pregnancy Following Spontaneous Conception: A Case Report and Literature Review. J. Clin. Gynecol. Obstet..

[3] Panelli D.M., Phillips C.H., Brady P.C. (2015). Incidence, diagnosis and management of tubal and nontubal ectopic pregnancies: A review. Fertil. Res. Pract..

[4] Doroszewska K., Milewicz T., Bereza T., Horbaczewska A., Komenda J., Kłosowicz E., Jach R. (2019). Cesarean scar pregnancy—Various methods of treatment. Folia Medica Cracoviensia.

[5] Ramadan M.K., Ramadan K., El Tal R., Salem Wehbe G.R., Itani S., Badr D.A. (2020). How safe is high-order repeat cesarean delivery? An 8-year single-center experience in Lebanon. J. Obstet. Gynaecol. Res..

[6] World Health Organization, Human Resource Planning (2015). WHO Statement on Caesarean Section Rates.

[7] Häger R.M., Daltveit A.K., Hofoss D., Nilsen S.T., Kolaas T., Øian P., Henriksen T. (2004). Complications of cesarean deliveries: Rates and risk factors. Am. J. Obstet. Gynecol..

[8] Maheux-Lacroix S., Li F., Bujold E., Nesbitt-Hawes E., Deans R., Abbott J. (2017). Cesarean scar pregnancies: A systematic review of treatment options. J. Minim. Invasive Gynecol..

[9] Petersen K.B., Hoffmann E., Larsen C.R., Nielsen H.S. (2016). Cesarean scar pregnancy: A systematic review of treatment studies. Fertil. Steril..

[10] Karampelas S., Salem Wehbe G., de Landsheere L., Badr D.A., Tebache L., Nisolle M. (2021). Laparoscopic isthmocele repair: Efficacy and benefits before and after subsequent cesarean section. J. Clin. Med..

[11] Fu P., Zhou T., Cui P., Wang W., Wang S., Liu R. (2022). Selection of Laparoscopy or Laparotomy for Treating Cesarean Scar Pregnancy: A Retrospective Study. Int. J. Gen. Med..

[12] Wang Y.L., Weng S.S., Huang W.C., Su T.H. (2014). Laparoscopic management of ectopic pregnancies in unusual locations. Taiwan. J. Obstet. Gynecol..

[13] Wang H.Y., Zhang J., Li Y.N., Wei W., Zhang D.W., Lu Y.Q., Zhang H.F. (2013). Laparoscopic management or laparoscopy combined with transvaginal management of type II cesarean scar pregnancy. J. Soc. Laparoendosc. Surg..

[14] Hudeček R., Felsingerová Z., Felsinger M., Jandakova E. (2014). Laparoscopic treatment of cesarean scar ectopic pregnancy. J. Gynecol. Surg..

[15] Jordans I.P., Verberkt C., De Leeuw R.A., Bilardo C.M., Van Den Bosch T., Bourne T., Brölmann H.A., Dueholm M., Hehenkamp W.J., Jastrow N. (2022). Definition and sonographic reporting system for Cesarean scar pregnancy in early gestation: Modified Delphi method. Ultrasound Obstet. Gynecol..

[16] Timor-Tritsch I.E., Monteagudo A., Cali G., El Refaey H., Agten A.K., Arslan A.A. (2016). Easy sonographic differential diagnosis between intrauterine pregnancy and cesarean delivery scar pregnancy in the early first trimester. Am. J. Obstet. Gynecol..

[17] Donnez O., Donnez J., Orellana R., Dolmans M.M. (2017). Gynecological and obstetrical outcomes after laparoscopic repair of a cesarean scar defect in a series of 38 women. Fertil. Steril..

[18] Vervoort A.J., Vissers J., Hehenkamp W.J., Brölmann H.A., Huirne J.A. (2018). The effect of laparoscopic resection of large niches in the uterine caesarean scar on symptoms, ultrasound findings and quality of life: A prospective cohort study. BJOG Int. J. Obstet. Gynaecol..

[19] Dindo D., Cuesta M., Bonjer H. (2014). The Clavien–Dindo Classification of Surgical Complications. Treatment of Postoperative Complications after Digestive Surgery.

[20] Kathopoulis N., Chatzipapas I., Samartzis K., Theodora M., Lardou I., Protopapas A. (2021). Laparoscopic management of cesarean scar pregnancy: Report of two cases with video-presentation of different operative techniques and literature review. J. Gynecol. Obstet. Hum. Reprod..

[21] Russell J.A., Gordon A.C., Williams M.D., Boyd J.H., Walley K.R., Kissoon N. (2021). Vasopressor Therapy in the Intensive Care Unit. Semin. Respir. Crit. Care Med..

[22] Lee G.G., Baek S.Y., Woo Kim T., Jeong C.Y., Ryu K.H., Park D.H. (2018). Cardiac arrest caused by intramyometrial injection of vasopressin during a robotic-assisted laparoscopic myomectomy. J. Int. Med. Res..

[23] Song L.P., Feng S.M., Jiang X.Q. (2021). Intramyometrial injection of vasopressin resulting in severe bradycardia during myomectomy. Chin. Med. J..

[24] Iannone P., Nencini G., Bonaccorsi G., Martinello R., Pontrelli G., Scioscia M., Nappi L., Greco P., Scutiero G. (2019). Isthmocele: From risk factors to management. Rev. Bras. Ginecol. Obs..

[25] Wang G., Liu X., Bi F., Yin L., Sa R., Wang D., Yang Q. (2014). Evaluation of the efficacy of laparoscopic resection for the management of exogenous cesarean scar pregnancy. Fertil. Steril..

[26] Pan Y., Liu M.B. (2017). The value of hysteroscopic management of cesarean scar pregnancy: A report of 44 cases. Taiwan. J. Obstet. Gynecol..

[27] Kremer T.G., Ghiorzi I.B., Dibi R.P. (2019). Isthmocele: An overview of diagnosis and treatment. Rev. Assoc. Méd. Bras..

[28] Vissers J., Hehenkamp W., Lambalk C.B., Huirne J.A. (2020). Post-Caesarean section niche-related impaired fertility: Hypothetical mechanisms. Hum. Reprod..

[29] Verberkt C., Lemmers M., de Leeuw R.A., van Mello N.M., Groenman F.A., Hehenkamp W.J.K., Huirne J.A.F. (2022). Effectiveness, complications, and reproductive outcomes after cesarean scar pregnancy management: A retrospective cohort study. AJOG Glob. Rep..

[30] Zhang X., Pang Y., Ma Y., Liu X., Cheng L., Ban Y., Cui B. (2020). A comparison between laparoscopy and hysteroscopy approach in treatment of cesarean scar pregnancy. Medicine.

